# MYC as a therapeutic target for the treatment of triple-negative breast cancer: preclinical investigations with the novel MYC inhibitor, MYCi975

**DOI:** 10.1007/s10549-022-06673-6

**Published:** 2022-07-30

**Authors:** Minhong Tang, Shane O’Grady, John Crown, Michael J. Duffy

**Affiliations:** 1 UCD School of Medicine, Conway Institute of Biomolecular and Biomedical Research, University College Dublin, Dublin, Ireland; 2 Department of Medical Oncology, St Vincent’s University Hospital, Dublin, Ireland; 3 Clinical Research Centre, St Vincent’s University Hospital, Elm Park, Dublin, D04 T6F4 Ireland

**Keywords:** MYC, Inhibitor, Breast cancer, Triple-negative, Treatment, MYCi975

## Abstract

**Background:**

MYC is one of the most frequently altered driver genes in triple-negative breast cancer (TNBC). The aim of this study was to evaluate targeting MYC for the treatment of TNBC.

**Methods:**

The anti-proliferative and apoptosis-inducing effects of the recently discovered MYC inhibitor, MYCi975 were investigated in a panel of 14 breast cancer cell lines representing the main molecular forms of breast cancer.

**Results:**

IC50 values for growth inhibition by MYCi975 varied from 2.49 to 7.73 µM. Response was inversely related to endogenous MYC levels as measured by western blotting (*p* = 0.047, *r* = − 0.5385) or ELISA (*p* = 0.001, *r* = − 0.767), i.e., response to MYCi975 decreased as endogenous MYC levels increased. MYCi975 also induced variable levels of apoptosis across the panel of cell lines, ranging from no detectable induction to 80% induction. Inhibition of proliferation and induction of apoptosis were greater in TNBC than in non-TNBC cell lines (*p* = 0.041 and *p* = 0.001, respectively). Finally, combined treatment with MYCi975 and either paclitaxel or doxorubicin resulted in enhanced cell growth inhibition.

**Discussion:**

Our findings open the possibility of targeting MYC for the treatment of TNBC. Based on our results, we suggest that trials use a combination of MYCi975 and either docetaxel or doxorubicin and include MYC as a putative therapy predictive biomarker.

**Supplementary Information:**

The online version contains supplementary material available at 10.1007/s10549-022-06673-6.

## Introduction

Although the treatment of patients with breast cancer has greatly improved in recent years [1], the form of breast cancer commonly referred to as triple-negative breast cancer (TNBC) continues to have a relatively poor prognosis [2]. TNBC is so called due to it’s lack of estrogen receptors (ER), progesterone receptors (PR) and HER2 [2]. Although constituting only about 15% of all invasive breast cancers, TNBC is responsible for a disproportionately high rate of deaths from the disease [3, 4]. This poor outcome is at least partially due to the lack of effective targeted therapies [1]. One of the reasons for the failure to find an effective targeted therapy for TNBC has been the widely held assumption that this subform of breast cancer lacks a highly prevalent driver gene that could be exploited for therapeutic purposes.

The c-MYC oncogene is one of the best studied cancer driver genes, promoting tumorigenesis via diverse mechanisms such as stimulating cell proliferation, blocking apoptosis, altering metabolism and depressing host immunity [5–7]. Overall, MYC is believed to be dysregulated in approximately 70% of cancers [5] Deregulation is mediated by multiple different mechanisms including gene amplification, gene translocation, altered methylation and enhanced intracellular signaling [6–8].

In breast cancer, MYC is the most frequently amplified gene [9, 10], with amplification occurring in 15–40% of cases [11–13]. The proportion of patients with amplification and/or overexpression of MYC is known to depend on the molecular subtype. We previously reported that MYC was more frequently amplified and exhibited greater expression at both the mRNA level and protein levels in the basal subtype compared with the other subtypes [14]. Most basal breast cancers (75 to 80%) possess the triple-negative (TN) phenotype [2]. Suggestive evidence that MYC may be playing a role in basal/TNBC progression was our finding that high levels of the oncoprotein correlated with adverse outcome in patients with this subtype [14]. MYC may thus be a driver gene for basal/TNBC and consequently may be a target for the development of new drugs to treat this subform of breast cancer.

Targeting MYC for cancer treatment, however, has proved difficult as the protein lacks a suitable site for high-affinity binding of low-molecular-weight inhibitors [15, 16]. Because of this, MYC has in the past been referred to as “undruggable” [15, 16]. The concept of MYC as an “undruggable” protein however, has recently changed as a result of the discovery of several promising MYC inhibitors [8, 17]. Indeed, one of the most promising MYC inhibitors, Omomyc [17], recently commenced evaluation in a clinical trial [18]. Another promising MYC inhibitor, known as MYCi975, act by blocking the interaction of MYC with its obligate partner, MAX [19]. This blockage leads to increased MYC degradation, impaired MYC-mediated gene expression, and suppressed tumor growth in vivo [19]. Furthermore, treatment of a prostate cancer model with MYCi975 was found to alter the tumor microenvironment, increase uptake of specific immune cells and enhance response to immunotherapy [19]. The aim of the current investigation was to investigate the potential of MYCi975 in the treatment of patients with breast cancer, with a particular focus on TNBC.

## Materials and methods

### Cell culture, viability and apoptosis assays

The origin and maintenance of the breast cancer cell lines used was as previously described [14, 20]. The cytotoxic drugs, docetaxel and doxorubicin were purchased from Sigma-Aldrich and Bio-techne, respectively while the survivin inhibitor YM-155 was obtained from Merck Life. The MYC antagonist MYCi975 (HY-129601) was purchased from MedChemtronica AB, Bergkällavägen 37C, 192 79 Sollentuna, Sweden. MYCMI-6 was a generous gift provided by Drs Alina Castell and Lars-Gunnar Larsson, Department of Microbiology, Tumor and Cell Biology, Karolinska Institutet, Stockholm, Sweden.

Cell growth was assessed using 3-(4,5-dimethylthiazol-2-yl)-2,5-diphenyltetrazolium bromide (MTT) (Sigma-Aldrich) as previously described [14, 20]. For measuring apoptosis, cells were seeded in 6-well plates at a density of 2 × 10^5^ per well and incubated overnight at 37 °C. Cells were then treated with dimethyl sulfoxide (DMSO) or 10 μM MYCi975. Following 48 h of incubation, they were harvested and stained with annexin-V and propidium iodide using the Annexin-V-FITC Apoptosis Detection Kit (Invitrogen). FACS analysis was performed using a BD FACSCanto ™.

### Western blot analysis

Following treatment with MYCi975 or MYCMI6, cells were lysed in RIPA buffer (Sigma-Aldrich) containing 0.1% of Triton X-100 (Sigma-Aldrich) as well as protease and phosphatase inhibitors (Roche). Following 20 min incubation on ice, cell lysates were centrifuged at 21,000 g for 20 min at 4 °C. The resultant supernatants were collected and stored at − 80 °C. Extracted proteins (70 or 100 μg) were then separated on a 10% or 12% handmade SDS-PAGE or precast Bolt Bis–Tris gels (Invitrogen) and transferred onto a PVDF membrane (Cytiva) [21]. Membranes were then blocked in 5% milk TBST for one hour at room temperature followed by incubation with the primary antibody: anti-c-Myc (Abcam, ab32072) or anti-survivin (Cell Signalling Technology, #2808 s). GAPDH (Merck Millipore or PROTEINTECH EUR, 6000–4-Ig) was used as loading control. After washing three times (10 min each) in TBST, membranes were immersed in 5% milk TBST containing HRP-conjugated secondary antibody (Santa Cruz) for 1.5 h at room temperature, followed by another three washes. Bands were developed by Super Signal chemiluminescence (ECL) (Thermo Fisher Scientific) and visualized using the Odyssey Imaging System (LI-COR Biosciences). Protein band intensities were quantified by densitometry using Image J software.

### Proteasome inhibition and protein half-life determination

For determining proteasome inhibition, cells were incubated with 10 µM MG132 (Sigma-Aldrich) for 22 h, followed by another 2 h incubation with 10 µM MYCi975 or DMSO. The 2 h incubation period with MYCi975 was chosen based on the results shown in Suppl Fig. 1, where degradation of survivin was clearly found after this time. For determining protein half-life, cells were treated with 10 µM MYCi975 or DMSO for 6 h and then incubated with cycloheximide (100 µg/ml). Cells were then harvested at the indicated time points and the relative levels of MYC were determined by western blotting.

### Enzyme-linked immunosorbent assay (ELISA)

c-MYC protein was quantified by ELISA using a c-Myc (Total) Human ELISA Kit, according to the manufacturer’s instructions (Invitrogen, KHO2041). Absorbance was measured on a plate reader at 450 nm (Multiscan Ascent, Labsystems).

### RNA extraction and real time PCR

RNA was extracted from cell lines using Trizol reagent (Sigma-Aldrich). One μg of RNA was reverse transcribed into cDNA using the High-Capacity cDNA Reverse Transcription Kit (Thermo Fisher Scientific). Survivin primers were supplied by Sino Biological Inc. GAPDH (Sigma-Aldrich) was used as a housekeeping-gene control. The amplification process was performed with the Roche Light Cycler 480, according to the manufacturer’s instructions. Changes in gene expression were calculated using the ddCt method.

### Statistical analysis

All the raw data were analyzed using Microsoft Excel 2016. Graph Pad Prism 5 was used to graph the calculated data points and calculate statistical values. The significance of data were evaluated using the Student’s unpaired, two-tailed t-test. Spearman’s rank correlation was used to assess the correlation between two groups. Combination Index (CI values) was calculated using CalcuSyn software (Biosoft). CI < 1 at 50% or 90% inhibition was used to indicate synergism [22].

## Results

### Effects of MYCi975 on the proliferation of human breast cancer cell lines

**Fig. 1:**
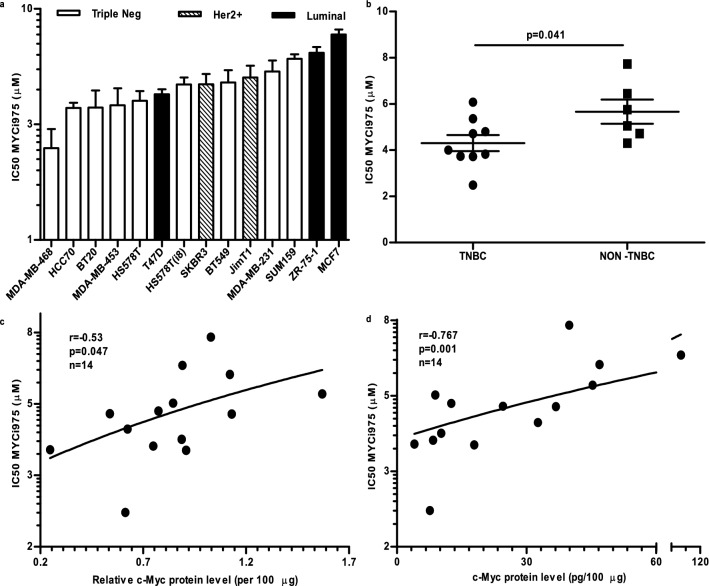
Effect of MYCi975 on proliferation of human breast cancer cells **a** Anti-proliferative effect of MYCi975 was measured in a panel of 14 breast cancer cell lines by MTT assay following 5 days incubation. IC50 values were then calculated and graphed using GraphPad Prism 5. **b** Scatter plot showing the relationship between MYCi975 IC50 values, and triple-negative (TN) status of cell lines. Scatter plots representation of the relationship between MYCi975 IC50 values, and c-Myc protein levels in cell lines, as determined by Western blotting **c** or by ELISA **d**. Data plotted are mean ± S.E.M (*n* = 3). Data were evaluated using the Student’s unpaired, two-tailed t-test

The effect of MYCi975 on cell proliferation was investigated using the MTT assay in a panel of 14 breast cancer cell lines, representative of the major molecular subtypes of this disease. Following 5 days incubation with increasing concentrations of MYCi975, the IC50 values for inhibition of growth varied from 2.49 to 7.73 µM (Fig. 1a). As our primary aim was to investigate MYCi975 as a potential inhibitor for TNBC, we compared the IC50 values in the TN versus the non-TN cell lines. As shown in Fig. 1b, TN cell lines were significantly more sensitive to growth inhibition with MYCi975 than non-TNBC cells (*p* = 0.041).

### Relationship between the growth inhibitory effects of MYCi975 and c-Myc protein expression levels of cell lines

As MYCi975 acts by binding to MYC [19], we related our IC50 values to endogenous MYC levels. Using both western blotting and ELISA to detect MYC, we found that the IC50 values for MYCi975 significantly increased with increasing endogenous MYC levels: using western blotting, *p* = 0.047, *r* = -0.5385, *n* = 14; using ELISA, *p* = 0.001, *r* = -0.767, *n* = 14 (Figs. 1c and 1d, respectively). Thus, the lower the cellular levels of MYC, the lower the IC50 value, i.e., a stronger the inhibitory response.

### Effects of MYCi975 on the induction of apoptosis

**Fig. 2:**
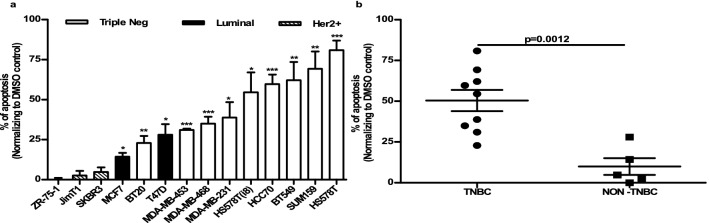
Effect of MYCi975 on apoptosis induction in human breast cancer cells **a** Effect of apoptosis induced by MYCi975 was analyzed in a panel of 14 breast cancer cell lines by flow cytometry, following 48 h incubation. % of apoptosis was then calculated and graphed using GraphPad Prism 5. * *p* < 0.05; ** *p* < 0.01 and *** *p* < 0.001. Data plotted are mean ± S.E.M (*n* = 3). **b** Scatter plot representation of the relationship between % of apoptosis induced by MYCi975 and triple-negative (TN) status of cell lines. Data was evaluated using the Student’s unpaired, two-tailed t-test.

To establish if the inhibitory effect of MYCi975 on cellular growth was mediated via induction of apoptosis, we investigated the effects of the inhibitor on this process in the same 14 breast cancer cell lines. The extent of induction of apoptosis varied from no detectable induction in ZR-75–1, JIMT1 and SKBR3 cell lines to approximated 80% induction in HS578T cells following 48 h incubation (Fig. 2a). Consistent with our results on inhibition of proliferation, the extent of apoptosis caused by MYCi975 was also significantly greater in the TN compared to the non-TN cells lines (*p* = 0.001; Fig. 2b).

### Effect of MYCi975 on levels of the anti-apoptotic protein survivin

**Fig. 3:**
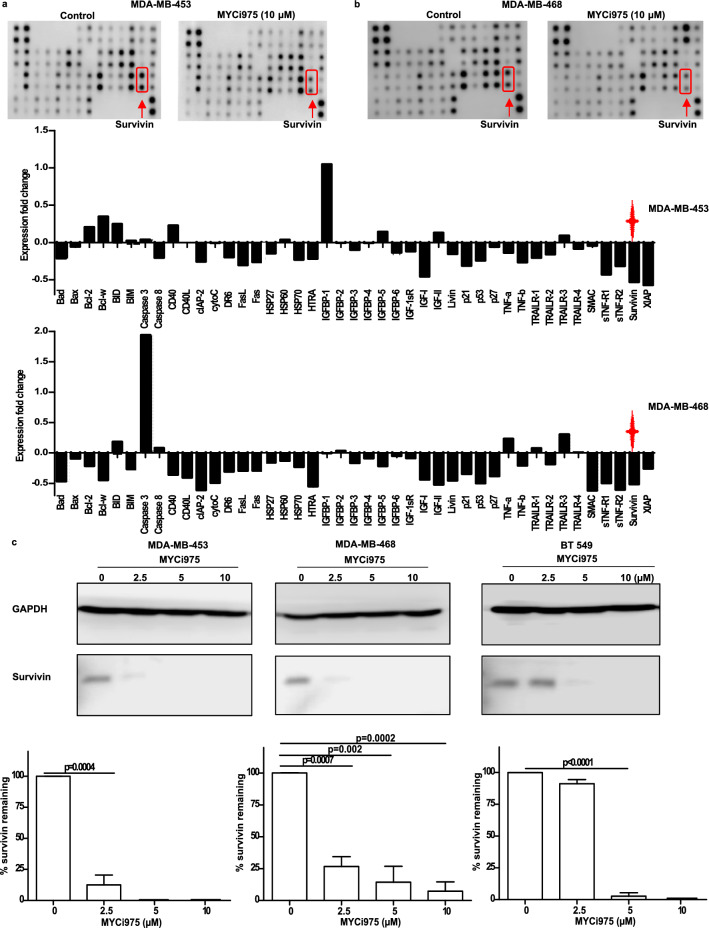
Effect of MYCi975 on degradation of survivin **a** MDA-MB-453 and **b** MDA-MB-468 cells. Cells were incubated with DMSO or 10 μM of MYCi975 for 48 h. Harvested cell lysates were analyzed by commercial human apoptosis membranes. **c** Following 48 h of treatment with MYCi975, MDA-MB-453, MDA-MB-468 and BT549 cell lysates were assessed by Western blotting using an antibody against survivin. GAPDH was detected as loading control. Expression fold changes and % of survivin remaining were then calculated and graphed using GraphPad Prism 5. Data plotted are mean ± S.E.M (*n* = 3) and evaluated using the Student’s unpaired, two-tailed t*-*test

To investigate the possible mechanism(s) of apoptosis induced by MYCi975, we first tested its effect on 43 apoptosis-associated proteins using apoptosis antibody arrays (Human Apoptosis Antibody Array-Membranes; Abcam, ab134001). Figures 3a and b shows proteins that were either upregulated or downregulated in MDA-MB-453 and MDA-MB-468 cells following treatment with 10 µM MYCi975 for 48 h. One of the potentially relevant proteins whose concentration was decreased in both the cell lines investigated was the apoptosis inhibitor, survivin. To confirm this finding, we treated three TNBC cell lines (MDA-MB-453, MDA-MB-468 and BT549) with different concentrations of MYCi975 and measured survivin by western blotting. As shown in Fig. 3c, treatments with MYCi975 resulted in decreased survivin levels in all three cell lines. As survivin is a potent inhibitor of apoptosis, its downregulation may be at least partially responsible for mediating MYCi975-induced apoptosis.

### MYCi975 decreased survivin protein stability via the proteasome system

**Fig. 4:**
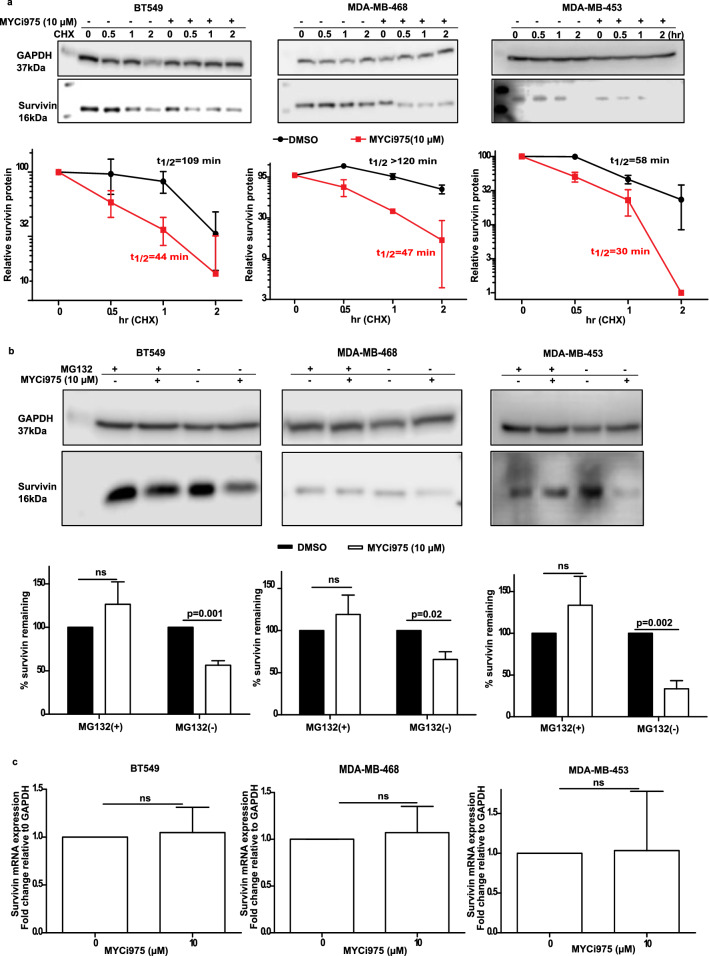
Effect of treatment with cycloheximide on MYCi975 mediated reduction of survivin protein stability **a** BT549, MDA-MB-468 and MDA-MB-453 were treated with DMSO or 10 µM of MYCi975 for 6 h, followed by incubation with cycloheximide (CHX) (100 μg/ml). Treated cells were then harvested at the indicated time points. **b** TNBC cells were incubated with 10 µM of MG132 for 22 h, followed by 2 h incubation with DMSO or 10 µM of MYCi975, before harvesting. Degradation of survivin were analyzed by Western blotting. **c** TNBC cells were incubated with DMSO or 10 μM of MYCi975 for 48 h. mRNA expression level of survivin in cells were determined by RT-PCR. Data were calculated and graphed using GraphPad Prism 5. Data plotted are mean ± S.E.M (*n* = 3) and evaluated using the Student’s unpaired, two-tailed t-test. In Fig 4a, line across DMSO and MYCi975 should be removed. In Fig 4b under MDA-MB-453, the GAPDH bands are invisible due to a dark overlay, please restore to that in our original submission

Theoretically, the downregulation of survivin could have occurred at an mRNA or protein level. To establish the mechanism of downregulation, we performed cycloheximide chase experiments [23]. As shown in Fig. 4, MYCi975 reduced the survivin protein half-life from 109 to 44 min in BT549 cells (Fig. 4a), from > 120 to 47 min in MDA-MB-468 cells (Fig. 4b) and from 58 to 30 min in MDA-MB-453 cells (Fig. 4c). To determine if this decreased stability was due to degradation by the proteasome system, we treated cells with the proteasome inhibitor, MG132. Following treatment with MG132, MYCi975-mediated degradation of survivin was reduced in all the three cell lines investigated (Fig. 4b), suggesting that the proteasome system is at least partially responsible for the decreased survivin stability. In contrast to our finding at the protein level, MYCi975 had no effect on the expression of survivin mRNA (Fig. 4c).

### Effects of MYCi975 in combination with the survivin inhibitor, YM-155 on cell viability

**Fig. 5:**
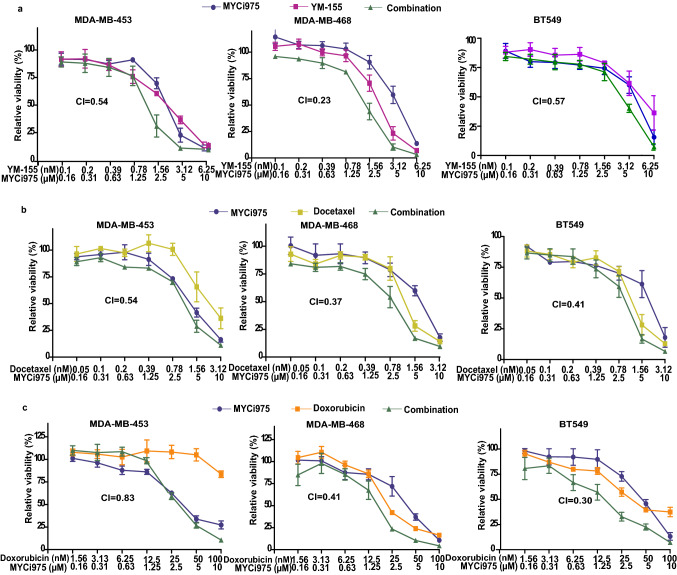
Effects of combined treatment with MYCi975 and other compounds on cell proliferation MDA-MB-453, MDA-MB-468 and BT 549 cells were treated with various concentrations of MYCi975 in combination with **a** YM-155, **b** docetaxel or **c** doxorubicin for five days. Cell growth was then measured by the MTT assay. Combination index (CI) values were calculated using Compusyn software. CI values < 1 indicate drug-synergy. Data were calculated and graphed using GraphPad Prism 5. Data plotted are mean ± S.E.M (*n* = 3).

As MYCi975 appears capable of altering survivn levels, we wanted to assess if combined inhibition of MYC and survivin could produce synergistic effects. We treated cell lines with MYCI975 and the survivin inhibitor, YM-155 and evaluated the potential for enhanced interaction between the two drugs using the Chou–Talalay method [22]. As shown in Fig. 5a, combined treatment resulted in synergistic growth inhibition in all three cell lines investigated.

### Effects of MYCi975 in combination with standard cytotoxic drugs on cell proliferation

To enhance the anti-proliferative effects of MYCi975, we combined it with two drugs widely used to treat breast cancer, i.e., docetaxel or doxorubicin. As shown in Fig. 5b, and c, the combination of MYCI975 and either of these drugs resulted in synergistic growth inhibition in the three different TN breast cancer cell lines studied.

### Relationship between response to MYCi975 and MYCMI-6

**Fig. 6:**
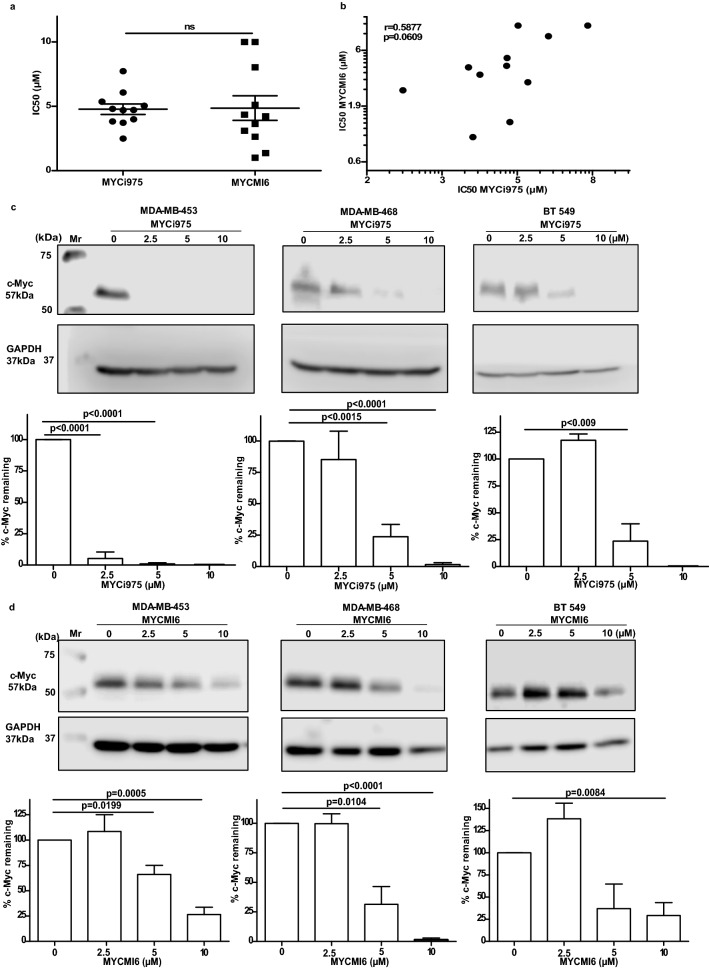
Relationship between MYCi975 and MYCMI6. **a** Comparative IC50 values for MYCi975 and MYCMI6 across 11 breast cancer cell lines. **b** Scatter plot showing the relationship between MYCi975 and MYCMI6 IC50 values. **c** Effect of MYCi975 on MYC degradation following incubation for 48 h. **d** Effect of MYCMI6 on MYC degradation following incubation for 48 h. Data plotted are mean ± S.E.M (*n* = 3) and evaluated using the Student’s unpaired, two-tailed t test

Similar with MYCi975, MYCMI-6 was previously reported to block the interaction between MYC and MAX and inhibit cell proliferation [14, 25]. Of the 14 cell lines investigated for inhibition of growth using MYCi975, 11 were also studied using MYCMI6. As shown in Fig. 6a, both inhibitors exhibited a similar range of IC50 values for growth inhibition. Despite having similar IC50 values, there was only a trend for a significant correlation between the IC50 values for the two compounds (Fig. 6b). Treatment with both compounds however, resulted in degradation of MYC (Fig. 6 c and d).

## Discussion

In this study, using a panel of 14 breast cell lines, we showed that the MYC inhibitor, MYCi975 reduced cell proliferation and promoted apoptosis in a cell line-dependent manner. In particular, the effect of MYCi975 on apoptosis was highly variable across the panel of cell lines investigated, ranging from effectively no induction of apoptosis in some cell lines to approximately 80% induction in others. Previously, Han et al. [19] reported that a close analog of MYCi975 (MYCi361) induced cleavage of caspase 3 in prostate cancer cells suggesting induction of apoptosis. However, these authors did not report the induction of apoptosis per se, although MYCi361 promoted immunogenic cell death and suppressed tumor cell growth [19].

A potential mechanism by which MYCi975 mediated apoptosis in our work was by reducing expression of survivin. Survivin is a member of the inhibitor of apoptosis (IAP) family of proteins which is believed to decrease apoptosis by inhibition of caspase activity, although the precise mechanism of this inhibition is unclear [25]. By decreasing survivin levels, MYCi975 could potentially play a role in enhancing apoptosis. Previous studies reported that MYC regulated survivin expression at the transcriptional level [26, 27]. In these studies, potential regulation at the protein level was not investigated. Our results clearly show that in the breast cancer lines studied in this investigation, that MYCi975 regulated survivin at the protein and not at the transcriptional levels.

Further evidence for a possible interaction between MYC and survivin was our finding of enhanced inhibition of cell proliferation when MYCi975 was combined with the survivin inhibitor, YM-155. We should state however, that our work does not exclude mechanisms other than a reduction in survivin levels by which MYCi975 might induce tumor cell apoptosis.

Furthermore, although YM155 has been clearly shown to reduce survivin expression, it may possess other activities [25], which might explain its ability to enhance the growth inhibitory or apoptosis-inducing actions of MYCi975. Indeed, it is also possible that the MYCi975-mediated reduction in survivin levels acted not only by enhancing apoptosis but also by inhibiting proliferation, as survivin has also been implicated in both these processes [25].

Previously, the MYC inhibitor, MYCMI-6 was also found to reduce cell proliferation in a diverse range of cancer cell lines [14, 24]. Despite both compounds inhibiting MYC via blocking the interaction between MYC and its partner MAX, the correlation between the IC50 values for the two compounds showed only a trend for significance. Although this lack of significance may be due to the relatively low number of cell lines investigated, it could also suggest that there are specific differences in the mechanisms by which the two compounds act.

As previously reported with MYCi975 [19], we also found that MYCMI-6 promoted degradation of MYC. Previously, Castell et al. [24] reported that MYCMI-6 failed to mediate degradation of MYC. The different results reported by Castell et al. [24] and our finding in this article may relate to different incubation periods with the MYC antagonist as Castell et al. [24] treated cells for 24 h, whereas we used a period of 48 h. Previously, another MYC inhibitor (Omomyc) was also shown to degrade MYC [28]. Thus, degradation of MYC protein appears to be mediated by multiple MYC antagonists. Although this effect is likely to be secondary to the inhibition of MYC-MAX interaction, it may also contribute to the anticancer activity of these compounds.

From a clinical point of view, a potentially important observation from our work was that the effects of MYCi975 on both proliferation reduction and apoptosis promotion was greater in TNBC cell lines than in non-TNBC cell lines. Furthermore, we found a significant inverse relation between cellular MYC levels and IC50 values for MYCi975, suggesting that MYC protein levels might be used as a predictive biomarker for response, assuming MYCi975 will enter clinical trials. In an earlier study, the MYCi975 analog, MYCi361 was found to exhibit a non-significant trend for an inverse relationship with MYC mRNA in the NCI60 panel of cells (*r* = − 0.23, *p* = 0. 086).

To enhance response, we investigated the growth inhibitory impact of MYCi975 in combination with two commonly used drugs to treat breast cancer, paclitaxel and doxorubicin. In the three cell lines investigated, synergistic growth inhibition was obtained with the MYCi975-cytotoxic drug combinations. These findings suggest that if MYCi975 entered clinical trials, it should be investigated in combination with either paclitaxel or doxorubicin.

In conclusion, we have shown that the novel MYC inhibitor MYCi975 decreased proliferation and induced apoptosis in breast cancer cell lines. The effect of MYCi975 on these two end points was significantly greater in TNBC than in non-TNBC breast cell lines. The growth inhibitory and apoptosis inducing effect of MYCi975 were significantly enhanced in the presence of docetaxel or doxorubicin. Thus, the combination of MYCi975 and paclitaxel or doxorubicin is a potential treatment for patients with TNBC. This therapy combination should now be investigated in a TNBC animal model system. Assuming our in vitro results can be confirmed in an animal model without major toxicity, the combination of MYCi975 and either docetaxel or doxorubicin should be considered for evaluation in a clinical trial in patients with TNBC.

## Supplementary Information

Below is the link to the electronic supplementary material.

Supplementary file1 (PDF 146 KB)

## Data Availability

Data will be shared on reasonable request to MT.
