# Supplementary figures and images for: MYC as a therapeutic target for the treatment of triple-negative breast cancer: preclinical investigations with the novel MYC inhibitor, MYCi975
Source: Breast Cancer Res Treat. 2022 Jul 30;195(2):105–15. doi: 10.1007/s10549-022-06673-6 (PMC9374613; doi:10.1007/s10549-022-06673-6)

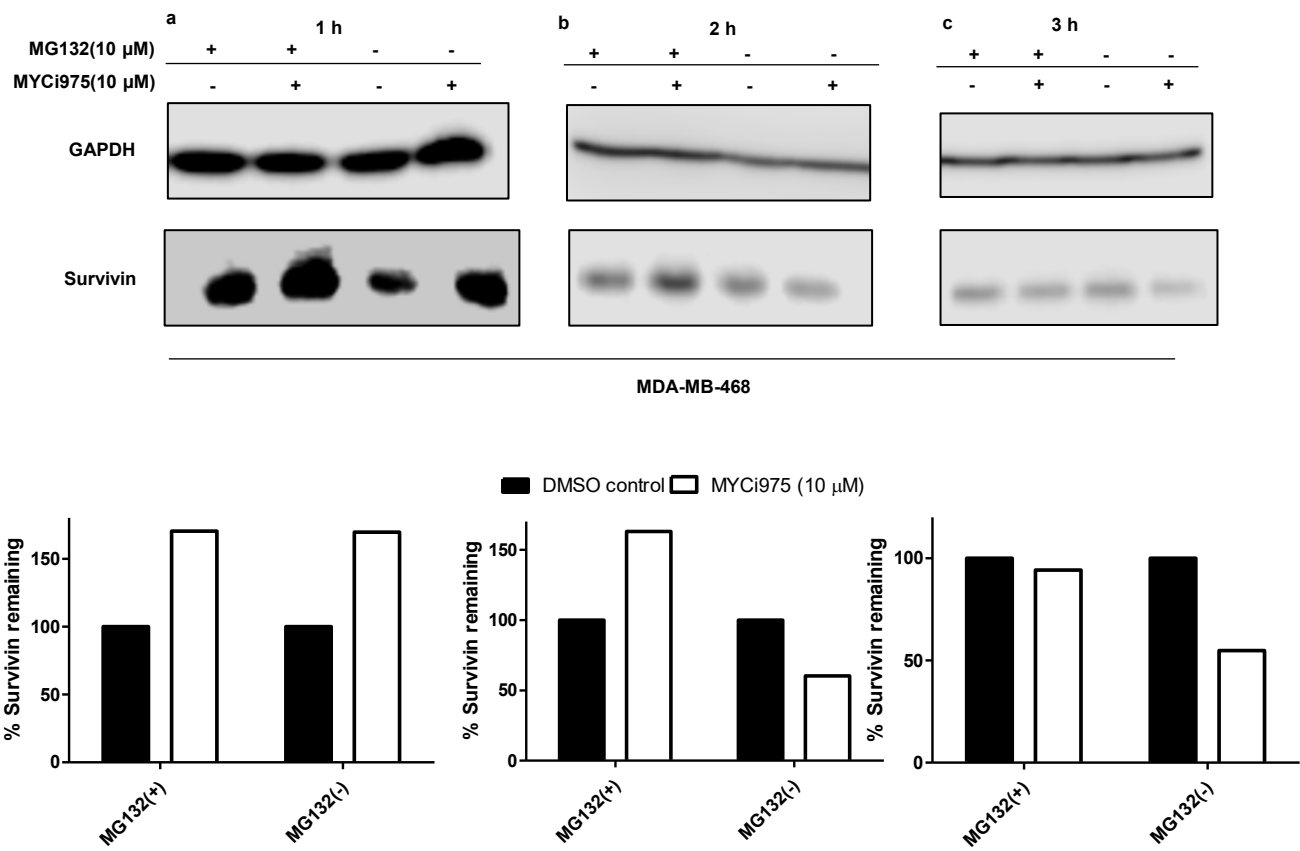

Supplement: Supplementary file 1 — Supplementary file1 (PDF 146 KB) [file 10549_2022_6673_MOESM1_ESM.pdf]
